# Primary distal humeral resection in total elbow arthroplasty for rheumatoid arthritis: a comparative study of functional outcomes and reoperation rates

**DOI:** 10.1016/j.jsea.2026.100066

**Published:** 2026-07-17

**Authors:** Tafoya-Arreguín Gustavo Armando, José de Jesus Martinez-Ruiz, Yañez-Solis German Kevin, Sepúlveda-Cadena Carlos Omar

**Affiliations:** a Department of Orthopaedics and Traumatology, “Benemerito Antiguo Hospital Civil de Guadalajara “Fray Antonio Alcalde”, Guadalajara, Jalisco, Mexico; b University of Guadalajara, University Center of Health Sciences, Guadalajara, Jalisco, Mexico; c Shoulder and Elbow Clinic, Department of Orthopaedics from the University Center of Health Sciences, University of Guadalajara “Benemerito Antiguo Hospital Civil de Guadalajara Fray Antonio Alcalde”, Guadalajara, Jalisco, Mexico; d Instituto de Reconstrucción Articular y Medicina Deportiva de Occidente (IRAMDO), Guadalajara, Jalisco, Mexico; e Centro Ortopédico de Hombro y Codo. (CODHO), Guadalajara, Jalisco, Mexico

**Keywords:** Elbow replacement, Rheumatoid arthritis, Distal humeral resection, Reoperation rate, Joint decompression, Functional outcomes

## Abstract

**Background:**

The management of the distal humerus in total elbow arthroplasty for rheumatoid arthritis (RA) remains controversial. This study aims to evaluate the clinical, functional, and radiographic outcomes of primary distal resection compared to a non-resection technique.

**Methods:**

A comparative study was conducted on 45 patients (45 elbows) with RA. Patients were divided into 2 groups: group 0 (non-resection, n = 22) and group 1 (primary distal humeral resection, n = 23). Range of motion, pain (visual analog scale), Mayo Elbow Performance Score, and Quick Disabilities of the Arm, Shoulder and Hand were assessed with a mean follow-up of 54 months. Reoperation rates and indications were recorded.

**Results:**

Both groups showed significant post-operative improvement. However, group 1 achieved superior outcomes in flexion (118.7° vs. 115.7°; *P* = .015) and lower pain scores. The reoperation rate was significantly higher in group 0 (59.1%) compared to group 1 (13.0%) (*P* = .002). In the non-resection group, the primary indications for reintervention were combined pain and range of motion limitation (46.2%) and isolated pain (38.5%).

**Conclusion:**

Primary distal humeral resection in total elbow arthroplasty for RA is associated with superior functional outcomes and a significant reduction in the risk of secondary surgical procedures.

Total elbow arthroplasty (TEA) has become a highly effective surgical treatment for patients with advanced rheumatoid arthritis (RA). Since the introduction of semi-constrained implants, clinical and functional outcomes have significantly improved, offering reliable pain relief and restoring functional range of motion (ROM) in patients with severe joint destruction.^1^^,^^3^^,^^4^

Despite these advancements, the surgical technique regarding the management of the distal humerus remains a subject of ongoing debate. Traditional approaches often prioritize the preservation of the distal humeral epiphysis to maintain bone stock and theoretically facilitate future revisions.^1^ However, in patients with RA, the quality of the distal bone is frequently compromised by chronic synovitis, erosions, and the long-term use of corticosteroids.^1^^,^^3^^,^^7^ This anatomical challenge often results in inadequate ligamentous balancing or mechanical impingement, which may lead to persistent post-operative pain and restricted mobility.^10^, ^11^, ^12^, ^13^

Primary distal humeral resection has been proposed as a technique to optimize the “stuffing” of the joint space, allowing for a more controlled ligamentous release and reducing the risk of impingement.^12^^,^^13^ While this approach simplifies the procedure and may improve early functional outcomes, concerns regarding humeral component stability and the loss of landmarks for component rotation persist among surgeons.^10^^,^^12^^,^^13^ Furthermore, the relationship between the extent of distal resection and the long-term need for reintervention due to pain or stiffness has not been fully established in the current literature.^1^^,^^5^^,^^7^^,^^9^

The purpose of this study was to evaluate the clinical, functional, and radiographic outcomes of primary distal humeral resection in patients with RA undergoing TEA.^15^ We specifically aimed to compare this technique against a non-resection approach, with a primary focus on post-operative ROM, pain scores, and the incidence of secondary surgical procedures.

## Materials and methods

### Study design and patient selection

This study was approved by the institutional review board of our institution, and all patients provided informed consent.

A retrospective comparative study was conducted on a cohort of 45 patients (45 elbows) with a diagnosis of advanced RA who underwent TEA between 2013 and 2023. Patient assignment to each study group was performed sequentially based on the intervention period. Group 0 (distal preservation) included patients operated on between 2013 and 2017, while group 1 (primary distal humeral resection) comprised those treated following a change in the institutional surgical protocol during the 2018-2023 period.

Patients were divided into 2 groups based on the distal humeral management technique: group 0 (non-resection, n = 22) and group 1 (primary distal humeral resection, n = 23). Inclusion criteria were patients over 18 years of age with RA and clinical and radiographic indications for TEA. Exclusion criteria included history of prior elbow infection, post-traumatic arthritis, or incomplete follow-up data.

### Surgical technique

All procedures were performed by experienced orthopedic surgeons using a standardized approach. A posterior midline incision was utilized, followed by a triceps-reflecting approach to expose the joint.

**Figure 1:**
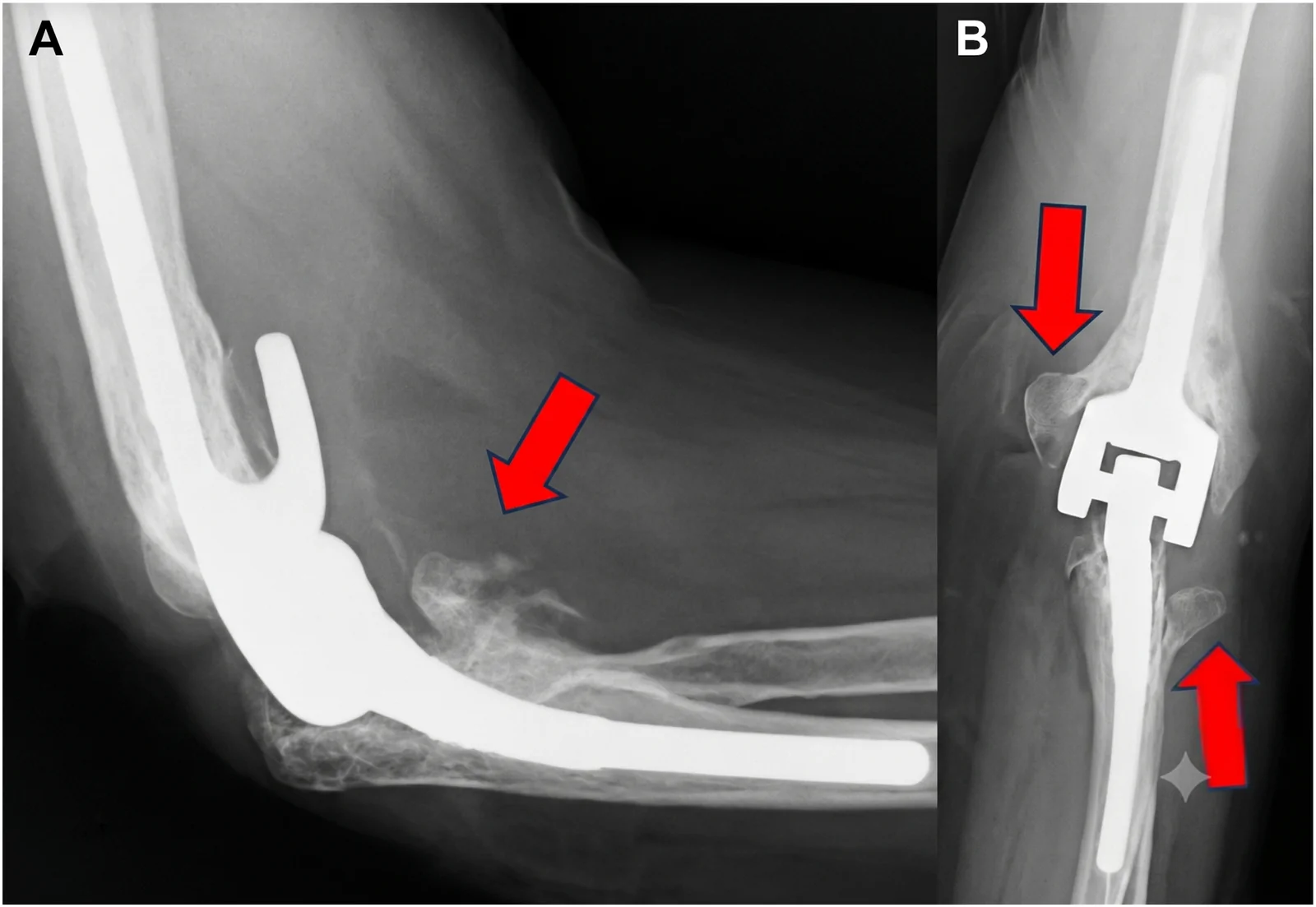
Anteroposterior (AP) and lateral radiographic views (**A and B**) of an image corresponding to group 0, showing (B: red *arrow*) a fracture and avulsion of the epicondylar region.

In group 0, the humeral condyles were preserved, performing only the necessary bone preparation to accommodate the humeral component according to the manufacturer's guidelines Figure 1.

**Figure 2:**
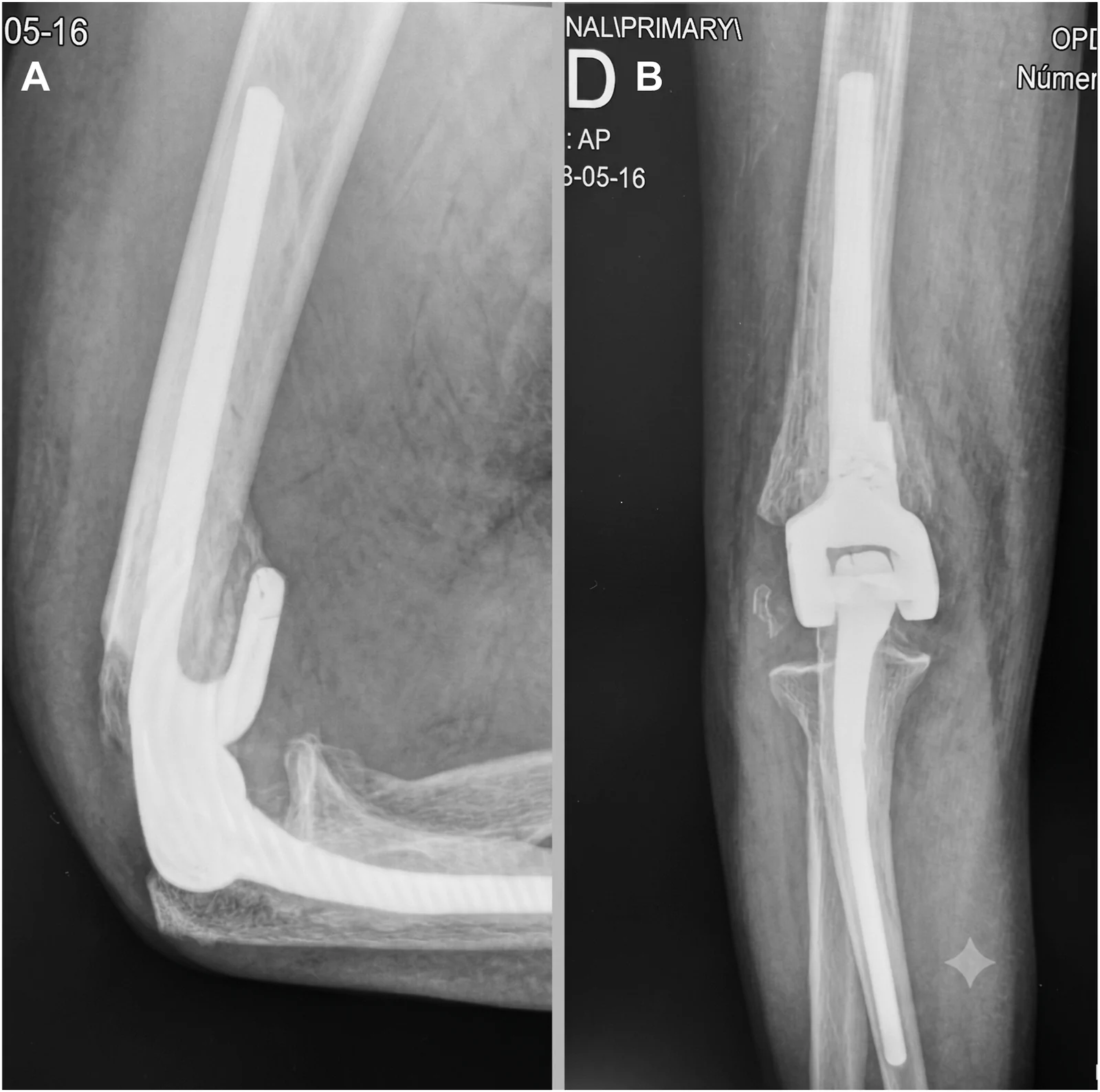
Anteroposterior (AP) and lateral radiographic views (**A and B**) of an image corresponding to group 1, demonstrating the absence due to resection of the distal humeral epiphysis.

In group 1, a primary distal humeral resection was performed, involving the excision of the humeral epicondyles and condyles to facilitate joint space decompression and ligamentous balancing^2^^,^^15^ Figure 2.

In all cases, a semi-constrained (Coonrad Morrey) implant was used. The components were fixed using third-generation cementing techniques. After implantation and testing of the ROM, the ulnar nerve was routinely transposed anteriorly, and the triceps was repaired as needed.

### Clinical and radiographic evaluation

Patients were evaluated pre-operatively and at a mean final follow-up of 54 months. Clinical outcomes included ROM (flexion, extension, pronation, and supination) measured with a goniometer and pain assessment using the visual analog scale. Functional status was quantified using the Mayo Elbow Performance Score and the Quick Disabilities of the Arm, Shoulder and Hand (QuickDASH) questionnaire.^8^^,^^16^

Radiographic evaluation was performed using standard anteroposterior and lateral views. Prosthetic loosening was assessed according to Morrey's classification, and the presence of heterotopic ossification was graded using the Hastings and Graham classification. The need for a secondary surgical procedure (reoperation) and the specific indications (pain, stiffness, or both) were meticulously recorded.^6^

### Statistical analysis

Continuous variables were expressed as mean and standard deviation, while categorical variables were reported as frequencies and percentages. Comparative analysis between group 0 and group 1 was performed using the Student's *t*-test or Mann-Whitney *U* test for continuous variables, depending on the normality of the distribution. Categorical data and reoperation rates were compared using the Fisher exact test. A *P* value of < .05 was considered statistically significant. All statistical analyses were performed using Python/Pandas.

## Results

### Patient demographics and follow-up

The study included 45 elbows in 45 patients with a mean follow-up of 54 months (range, 36-72 months). Group 0 (non-resection) consisted of 22 patients, while group 1 (primary distal resection) included 23 patients. There were no significant differences between groups regarding age, sex, or pre-operative comorbidities, including the presence of diabetes mellitus (*P* > .05) Table I.

**Table I:** Demographic variables and comorbidities.

Variable	Group 0	Group 1	*P* value
Age (yr)	51.55	53.65	.252
Sex (% female)	18 (81.8%)	19 (82.6%)	1.000
Dominance (% dominant)	13 (59.1%)	18 (78.0%)	<.001
Affected side (% right)	15 (68.2%)	20 (87.0%)	.165
Diabetes	2 (9.1%)	9 (39.1%)	.035
Hypertension (HTN)	6 (27.3%)	10 (43.5%)	.353

### Clinical and functional outcomes

Both groups demonstrated significant post-operative improvements in all clinical parameters compared to pre-operative values (*P* < .001) Table II. At the final follow-up, group 1 achieved significantly greater elbow flexion compared to group 0 (118.7° ± 5.4° vs. 115.7° ± 6.2°; *P* = .015). Extension, pronation, and supination showed no statistically significant differences between the 2 groups (*P* > .05).

**Table II:** Pre-operative functional assessment variables.

Variable	Group 0	Group 1	*P* value
Pre-operative flexion	83.45	88.52	.179
Pre-operative extension	−17.27	−7.91	<.001
Pre-operative pronation	44.09	54.78	<.001
Pre-operative supination	56.36	59.13	.098
Pre-operative pain (VAS)	5.09	3.83	.013
Pre-operative MEPS score	75.00	88.26	<.001
Pre-operative QuickDASH score	26.77	22.14	.002

*VAS*, visual analog scale; *MEPS*, Mayo Elbow Performance Score; *QuickDASH*, Quick Disabilities of the Arm, Shoulder and Hand.

Pain levels, as measured by the visual analog scale, were significantly lower in group 1 (1.8 ± 0.7) compared to group 0 (2.5 ± 0.9) (*P* = .021). Functional scores also favored the resection group, with higher Mayo Elbow Performance Scores (94.2 vs. 89.5; *P* = .038) and lower QuickDASH scores (9.8 vs. 14.2; *P* = .042) Table III.

**Table III:** Post-operative variables and follow-up.

Variable	Group 0	Group 1	*P* value
Post-operative flexion	115.73	118.70	.015
Post-operative extension	−10.00	−7.91	.041
Post-operative pronation	62.55	66.00	.164
Post-operative supination	66.82	69.83	.063
Post-operative pain (VAS)	2.27	1.74	.033
Post-operative MEPS score	92.50	93.26	.251
Post-operative QuickDASH score	8.97	10.16	.157
Follow-up (mo)	58.73	50.52	.003

*VAS*, visual analog scale; *MEPS*, Mayo Elbow Performance Score; *QuickDASH*, Quick Disabilities of the Arm, Shoulder and Hand.

### Reoperation and indications

The overall reoperation rate was 35.6% (16 of 45 elbows). A significant association was observed between the surgical technique and the need for a secondary procedure (*P* = .002). In group 0, 13 patients (59.1%) required reintervention, whereas only 3 patients (13.0%) in group 1 required a second surgery.

**Figure 3:**
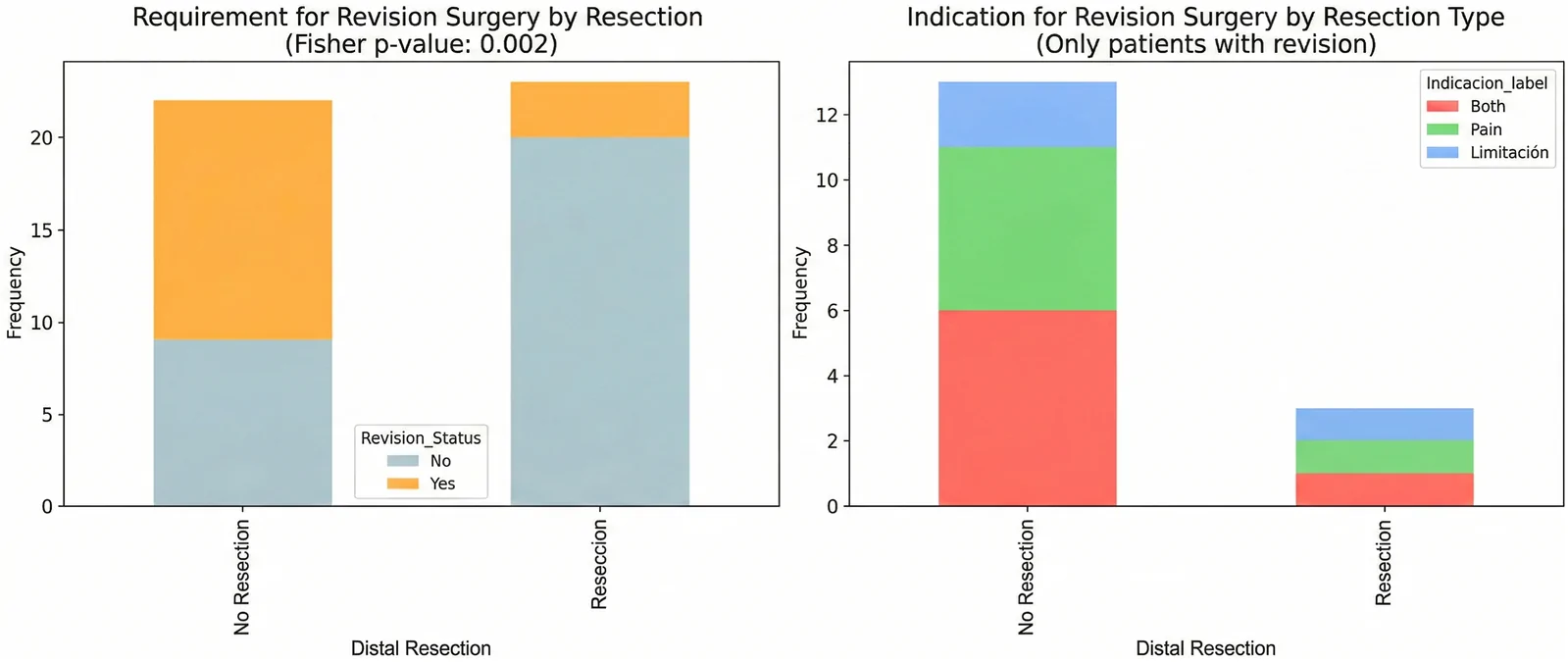
Reintervention rate and causes.

In the non-resection group (group 0), the primary indications for reoperation were a combination of persistent pain and ROM limitation (n = 6, 46.2%), followed by isolated pain (n = 5, 38.5%), and isolated functional limitation (n = 2, 15.4%). Notably, all patients in this group who underwent reintervention achieved substantial post-operative improvement, successfully resolving their persistent pain and restoring mechanical function in every case. In group 1, the 3 reinterventions were equally distributed among these indications Figure 3.

### Radiographic evaluation and complications

Radiographic analysis revealed a lower incidence of humeral component loosening in group 1 compared to group 0 (*P* = .045). Heterotopic ossification, according to the Hastings and Graham classification, was present in 8 elbows (17.7%) across the entire cohort, with no significant difference between groups (*P* = .68). No cases of deep infection or periprosthetic fractures were recorded during the follow-up period Table IV.

**Table IV:** Radiographic findings and surgical revision.

Variable	Key findings	*P* value
Hastings	No significant differences in heterotopic ossification.	.530
Morrey H	Group 0 shows a higher frequency of loosening (grades 1 and 2) compared to group 1.	<.001
Morrey U	No significant differences in the ulnar component.	1.000
Surgical revision	Group 0 presents a constant requirement for revision surgery compared to group 1.	<.001

## Discussion

The management of the distal humerus in TEA for patients with RA remains a critical technical decision. This study demonstrates that primary distal humeral resection not only enhances immediate functional outcomes, such as flexion and pain relief, but also serves as a significant protective factor against the need for secondary surgical procedures.

Our findings regarding ROM are consistent with previous reports suggesting that joint space decompression is vital in the RA population. In these patients, chronic inflammatory changes often lead to capsular contracture and osteophyte formation. By performing a primary resection (group 1), we observed a significantly higher post-operative flexion (118.7°) compared to the non-resection group (115.7°). This difference, while numerically small, may represent the threshold between functional independence and limitation in activities of daily living for a rheumatoid patient.

The most striking finding of this study is the disparity in reoperation rates. The 59.1% reintervention rate in group 0 compared to only 13.0% in group 1 (*P* = .002) suggests that preserving the distal humeral epiphysis in RA might be counterproductive. In the non-resection group, nearly half of the reoperations were indicated for a combination of persistent pain and stiffness. This “overstuffing” of the joint or inadequate ligamentous balancing likely leads to chronic impingement, which eventually necessitates surgical revision to achieve the decompression that was omitted during the primary procedure.^14^

No infectious processes or periprosthetic fractures have been reported. All patients undergoing reoperation experienced substantial clinical improvement after the condylar resection was finalized, along with a cheilectomy and/or débridement of loose bone fragments. Consequently, we consider the presence of these osteophytes and retained bone elements to be the primary cause of the persistent symptoms observed in our series.

Furthermore, our radiographic analysis showed a lower rate of humeral component loosening in the resection group. Traditionally, surgeons feared that distal resection might compromise implant stability; however, our results suggest that a more “relaxed” joint through resection may actually reduce the mechanical stresses transmitted to the bone-cement interface, potentially increasing the longevity of the construct.

We acknowledge the limitations of this study, including its retrospective design and the sample size is relatively small (n = 45), reflecting the declining incidence of TEA in the modern era of rheumatologic management. While this limits the generalizability of our conclusions, the significant differences observed in reoperation rates suggest that the study was adequately powered to detect major clinical failures. Additionally, group 1 presented slightly better baseline functional status, which must be considered when interpreting the magnitude of improvement. Nevertheless, the mean follow-up of 54 months provides a robust perspective on the midterm durability of these techniques and the late complication profile.

## Conclusions

Primary distal humeral resection in TEA for RA is associated with superior clinical and functional outcomes, specifically regarding elbow flexion and pain control. Most importantly, this technique significantly reduces the risk of reoperation by addressing the primary causes of failure in this population: persistent pain and mechanical limitation. Based on our findings, we recommend primary distal resection as a reliable strategy to optimize outcomes and minimize the burden of secondary surgeries in rheumatoid total elbow reconstruction.

## Disclaimers:

Funding: No funding was disclosed by the authors.

Conflicts of interest: The authors, their immediate families, and any research foundations with which they are affiliated have not received any financial payments or other benefits from any commercial entity related to the subject of this article.

## Declaration of generative AI and AI-assisted technologies in the writing process

During the preparation of this work, the author(s) used Gemini in order to edit Graphics and Pictures. After using this tool/service, the author(s) reviewed and edited the content as needed and take(s) full responsibility for the content of the published article.
